# Prevalence and associated factors of fear of progression in patients with heart failure in China: a scoping review

**DOI:** 10.3389/fpsyt.2026.1865683

**Published:** 2026-08-06

**Authors:** Lei Wang, Yuejiao Sun, Wentong Zuo, Qingya Han, Lina Yang, Chunhua Guo, Chunxia Zhou

**Affiliations:** 1 Nursing School, Hebei Medical University, Shijiazhuang, China; 2 Department of Cardiology II, Hebei Medical University Third Hospital, Shijiazhuang, China; 3 Department of Orthopedic Surgery, Hebei Medical University Third Hospital, Shijiazhuang, China

**Keywords:** associated factors, fear of progression, heart failure, prevalence, scoping review

## Abstract

**Introduction:**

Heart failure (HF) is a complex, multifactorial clinical syndrome with an increasing global prevalence and high rates of rehospitalization and mortality. Fear of progression (FoP) is a common psychological concern among patients with chronic diseases, such as cancer and diabetes. Given the progressive nature of HF, FoP may represent an equally important psychological concern in this population. It is associated with poor treatment adherence, reduced quality of life, and a higher risk of adverse cardiovascular events and unplanned hospital readmissions. However, studies specifically investigating FoP in patients with HF remain relatively limited, and the existing evidence has not yet been systematically synthesized. This scoping review aimed to map the assessment tools, prevalence, and associated factors of FoP among Chinese patients with HF.

**Methods:**

This scoping review was reported following the 2018 Preferred Reporting Items for Systematic Reviews and Meta-Analyses Extension for Scoping Reviews (PRISMA-ScR) guidelines. Relevant studies were searched in ten databases, including PubMed, Web of Science Core Collection, Scopus, Embase, CINAHL, CNKI, Wanfang, VIP, CBM, and CMA Journals Full-text Database, from database inception to December 31, 2025. The Joanna Briggs Institute (JBI) Critical Appraisal Checklists were used to assess the quality of included studies.

**Results:**

This scoping review identified 2,875 records, and 17 studies from China were finally included. These studies included 3,918 patients with HF, and the prevalence of FoP ranged from 23.40% to 48.00%. Twenty-nine associated factors were identified and grouped into four categories: sociodemographic factors, disease and treatment factors, psychological and cognitive factors, and health and functional factors.

**Conclusion:**

This scoping review found that FoP is relatively common in patients with HF in China and is associated with a range of factors. These findings may support early identification of high-risk patients and the development of integrated psychological and social care strategies in HF management. Future research should focus on developing assessment tools specific to HF, understanding how FoP changes over time, identifying effective interventions, and validating these findings across diverse geographical populations.

## 1 Introduction

Heart failure (HF) is a clinical syndrome caused by structural or functional abnormalities of the heart and is often characterized by dyspnea, fatigue, and edema (1, 2). Population-based studies indicate that the age-standardized global prevalence of HF rose to 676.68 per 100,000 population in 2021 and has increased over time (3). Recent estimates indicate that about 7.7 million people in the United States and nearly 14.3 million people in China are living with HF (4, 5). Furthermore, the one-year readmission rate among patients with HF may reach 35.7%, while the one-year mortality rate may reach 23.3% (6). As an incurable and progressive chronic disease, HF necessitates rigorous and lifelong self-management, which typically involves daily weight monitoring, fluid and sodium restriction, medication adherence, and the early recognition of decompensation (2). Despite these efforts, frequent acute exacerbations and repeated hospital readmissions, along with the progressive loss of autonomy, leave patients continuously exposed to illness uncertainty and functional decline (7, 8), imposing a severe psychological burden on patients with HF. A recent study exploring the emotional experiences of patients with HF further elucidates the complexity of this psychological distress (9). In long-term disease management, patients frequently experience helplessness and guilt driven by fear of symptom exacerbation and perceived family burden, leading some to hide their illness to avoid triggering family anxiety. Moreover, adverse life events such as bereavement, divorce, or comorbid severe illnesses often act as powerful emotional triggers. These incidents damage patients’ physical and mental states, heighten emotional fragility, and turn daily self-care into a persistent psychological burden. The combined physical and mental strain caused by these negative events can significantly impair cardiac function.

In recent years, the World Health Organization has urged greater attention to mental health, and many clinical guidelines have highlighted its close link to overall health (2, 10, 11). Fear of progression (FoP), a common adverse psychological response among people living with chronic illnesses such as cancer and diabetes, has received growing academic attention. The concept of FoP was first introduced by Dankert et al. in 2003 (12). At present, FoP is generally defined as a patient’s subjective perception of disease progression or recurrence and its related negative physical, psychological, and social effects (13); this disease-specific emotional state differentiates it from the broad clinical anxiety disorders outlined in the International Classification of Diseases, 11th Revision (ICD-11) (14). However, in the context of HF, FoP extends beyond a general fear of disease progression to encompass profound fears regarding worsening cardiac function, severe dyspnea, functional decline, loss of social roles, dependence on caregivers, and impending death. Importantly, growing evidence suggests that FoP is associated with several adverse outcomes, including reduced adherence to treatment, impaired physical function, reduced quality of life (15, 16), delayed return to work (17), increased psychological distress, and a higher risk of adverse cardiovascular events and unplanned hospital readmissions (18). Given the progressive nature of HF, FoP may represent an equally important psychological concern in this population. However, evidence on FoP in patients with HF remains limited, and existing studies vary in design, reported prevalence, and associated factors. These inconsistencies make it difficult to obtain a clear understanding of FoP in this population. Therefore, this scoping review aimed to map the available evidence on FoP assessment tools, prevalence, and associated factors among patients with HF. These findings may help healthcare professionals identify patients at high risk and implement targeted psychological interventions, while also supporting the development of FoP-specific assessment tools for this population.

## 2 Methods

This scoping review was conducted in accordance with the Arksey and O’Malley framework and reported following the 2018 Preferred Reporting Items for Systematic Reviews and Meta-Analyses Extension for Scoping Reviews (PRISMA-ScR) guidelines (19, 20). The protocol was registered with the Open Science Framework (OSF; https://doi.org/10.17605/OSF.IO/UY4KH).

### 2.1 Defining the research questions

Following the PCC framework (21), with the population defined as patients with HF, the concept as FoP, and the context as associated factors, three research questions were formulated: (1) Which validated tools are available to assess FoP in patients with HF? (2) What is the prevalence of FoP in patients with HF? (3) Which factors are associated with FoP severity in this population?

### 2.2 Inclusion and exclusion criteria

Studies were considered eligible if they met the following criteria: (1) the study population consisted exclusively of adult patients with confirmed HF; (2) FoP was assessed using a validated or clearly described measurement tool; (3) the study reported the prevalence, level, trajectory, or associated factors of FoP in patients with HF; (4) the study was an original quantitative study, including cross-sectional or cohort studies; and (5) the article was published in Chinese or English. Studies were excluded if they were duplicate publications or had incomplete or unavailable data.

### 2.3 Literature search strategy

A systematic literature search was carried out across multiple Chinese and English databases, including PubMed, Web of Science Core Collection, Scopus, Embase, CINAHL, China National Knowledge Infrastructure (CNKI), Wanfang, VIP (Chinese Scientific Journal Database), Chinese Medical Association Journals Full-text Database (CMA Journals Full-text Database), and Chinese Biomedical Literature Database (CBM). The search covered studies published from the start of each database to December 31, 2025. A combination of subject headings and free-text terms was used to develop the search strategy. After eligible studies were identified, citation snowballing was also performed by screening their reference lists. The search query was as follows (using PubMed as an example): (((fear[MeSH Terms]) OR ((Fear*[Title/Abstract]) OR (Cue, Threat[Title/Abstract]) OR (Threat Cue*[Title/Abstract]) OR (Threat Sensitivit*[Title/Abstract]) OR (Sensitivity, Threat[Title/Abstract]))) OR ((Fear of Recurrence[Title/Abstract]) OR (Fear of progression[Title/Abstract]) OR (Disease progression fear[Title/Abstract]) OR (FoP[Title/Abstract]) OR (Fear of Relapse[Title/Abstract]))) AND ((Heart Failure[MeSH Terms]) OR (Heart Failure[Title/Abstract]) OR (Cardiac Failure[Title/Abstract]) OR (Heart Decompensation[Title/Abstract]) OR (Congestive Heart Failure[Title/Abstract]) OR (Myocardial Failure[Title/Abstract])). Detailed search strategies for all databases are provided in the **Supplementary Material**.

### 2.4 Study selection and data extraction

All identified records were imported into Zotero 7.0 for duplicate removal. Two independent reviewers screened titles and abstracts against the predefined inclusion and exclusion criteria. The full texts of potentially eligible studies were then retrieved and assessed for final inclusion. Any disagreements between the two reviewers were resolved through discussion or by consulting a third reviewer. Data extraction was performed independently by two reviewers using a predesigned data charting form, with discrepancies resolved through discussion or consultation with a third reviewer, and data were extracted for the following variables: study characteristics, sample characteristics, assessment tool, FoP prevalence, and associated factors. Throughout the study, descriptive statistics and narrative reviews were used to organize and synthesize research findings, with thematic analysis utilized to categorize influencing factors (22).

### 2.5 Quality assessment

Quality appraisal was conducted to describe the methodological quality of the included studies rather than to exclude studies from the review. It was performed using the Joanna Briggs Institute (JBI) Critical Appraisal Checklists for cross-sectional and cohort studies (23). Each item was rated as “Yes”, “No”, “Unclear”, or “Not applicable”. “Yes” responses were given 1 point, while all others were given 0 points. The total score was converted to a percentage. Studies scoring 85% or above were classified as high quality, whereas those scoring 60% to 84% were considered moderate quality (24). The quality assessment was conducted independently by two reviewers, and any differences were addressed through discussion with a third reviewer.

## 3 Results

### 3.1 Study selection

**Figure 1:**
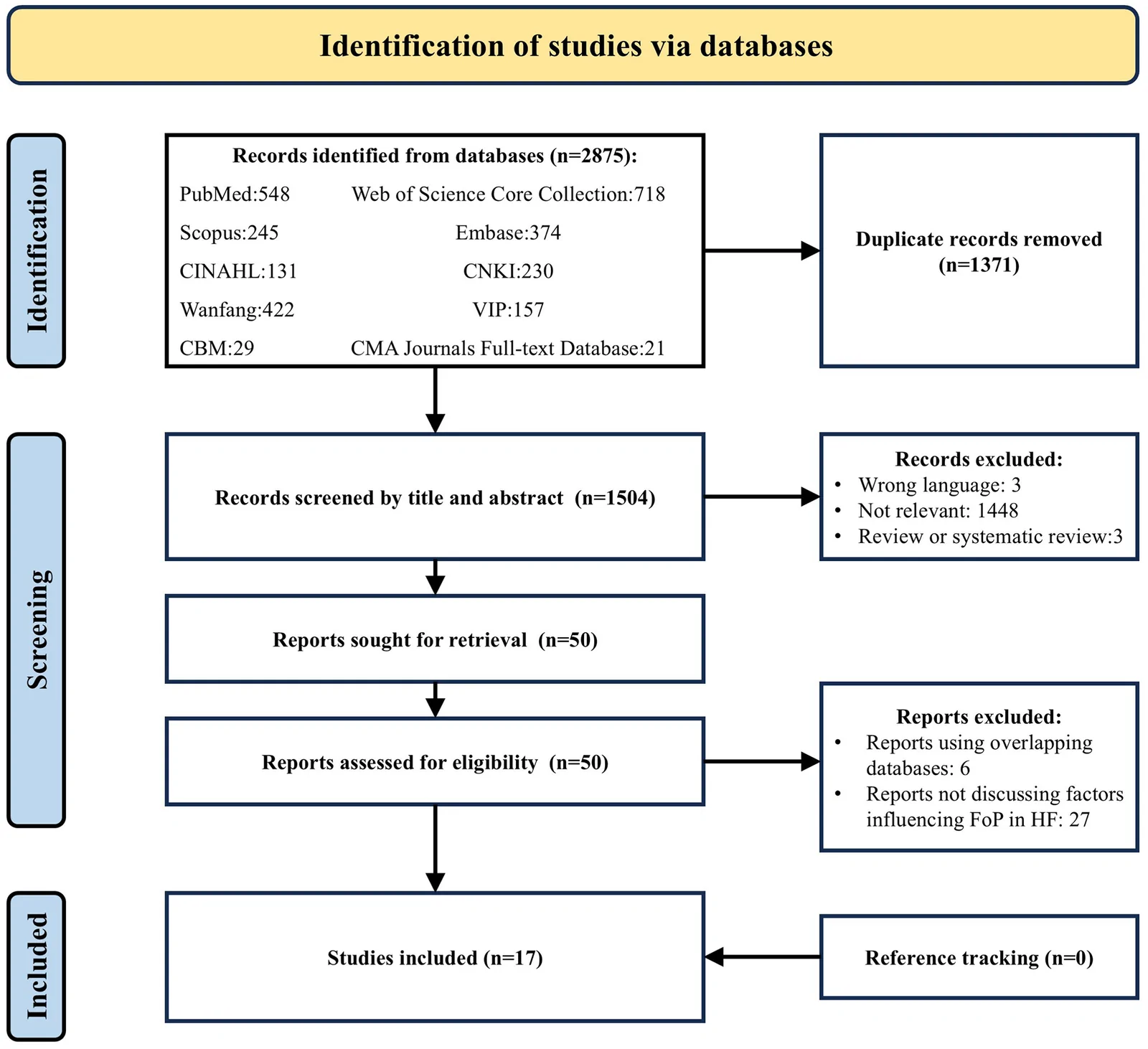
Flow chart of the search strategy and study selection process.

A systematic search was conducted across multiple databases from their inception to December 31, 2025, and the final search was conducted on January 31, 2026, identifying 2,875 records. After duplicates were removed and the titles, abstracts, and full texts were screened, 17 studies were finally included. Details are shown in **Figure 1**.

### 3.2 Study characteristics

**Figure 2:**
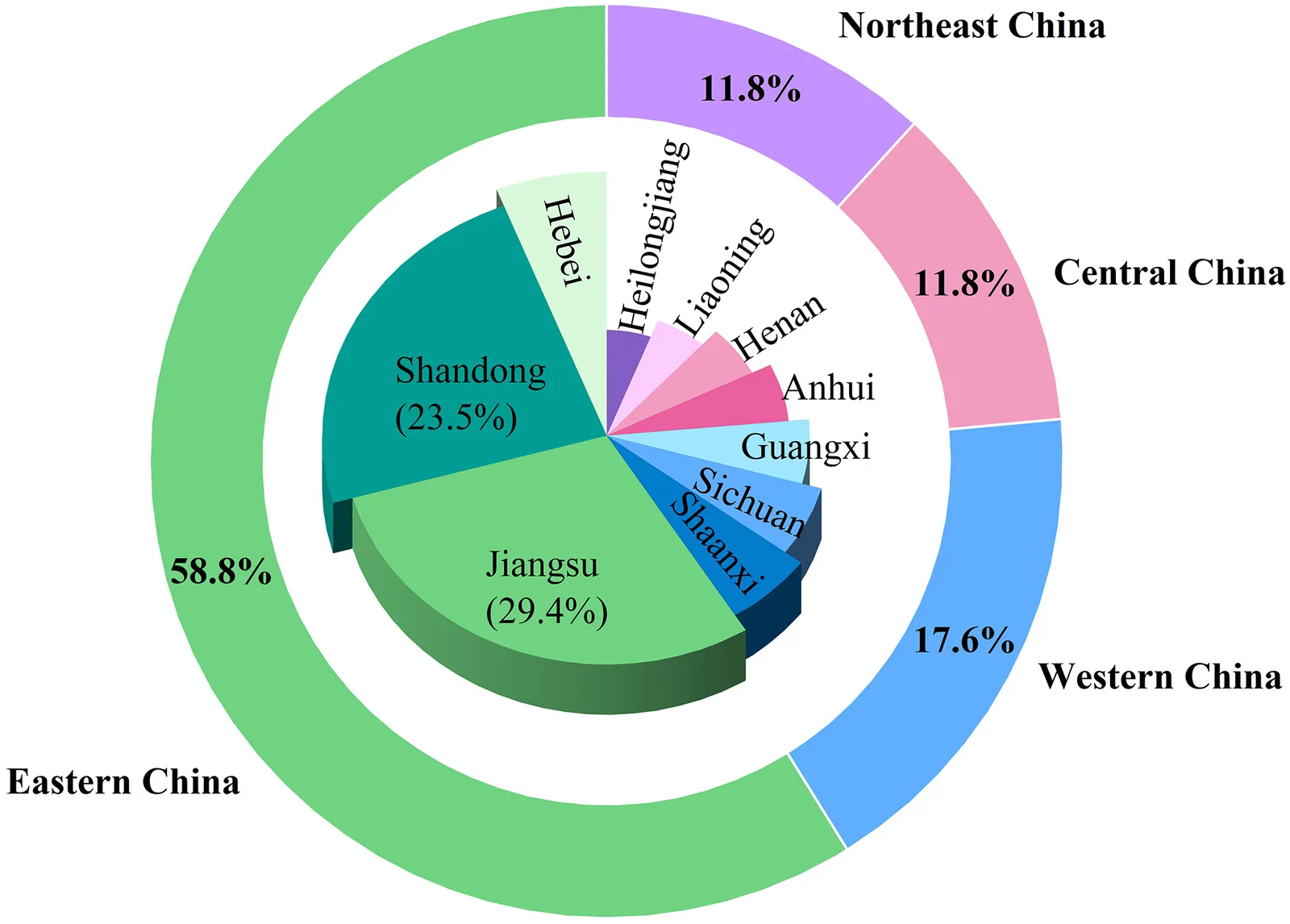
Distribution of study regions. The outer ring shows the categorization of the four major regions. The inner ring details the provinces included in this study. Specific proportions for Jiangsu and Shandong are labeled, while all other provinces are represented by equal, smaller segments.

This scoping review included 17 studies (18, 25–40), all conducted in China and published between 2021 and 2025 (**Table 1**; **Figure 2**). Of these, 14 were published in Chinese (18, 25–32, 36–40) and three in English (33–35). Regarding study design, 15 were cross-sectional studies (25–37, 39, 40) and two were cohort studies (18, 38). Ten were single-center studies (25–29, 35–38, 40), whereas seven were multicenter studies (18, 30–34, 39). Four studies enrolled only patients aged 60 years or older (27, 31, 34, 36), and one focused specifically on patients in the vulnerable phase of HF (30). Among the 17 included studies, 3,918 participants were included in total. Each study enrolled between 120 and 362 participants, and the proportion of female participants ranged from 33.19% to 52.43%.

**Table 1:** Characteristics of included studies.

Author	Study design	Year	Study region	Sample source	Age (years)	Sample size	Female (%)	Assessment tool	FoP score, mean ± SD	FoP prevalence (%)	Associated factors
Du et al. (25)	Cross-sectional study	2021	Heilongjiang, China	Single-center	≥18	150	43.33	FoP-Q-SF	31.89 ± 6.22	41.30	16;18
Cao Y et al. (26)	Cross-sectional study	2022	Jiangsu, China	Single-center	≥18	198	36.86	FoP-Q-SF	31.97 ± 8.96	44.44	9;11;16
Xiong et al. (18)	Cohort study	2023	Jiangsu, China	Multicenter	≥18	267	44.19	FoP-Q-SF	30.54 ± 6.53	34.20	1;12;19;20;25
Zhao et al. (27)	Cross-sectional study	2023	Jiangsu, China	Single-center	≥60	223	51.56	FoP-Q-SF	33.30 ± 7.00	48.00	5;8;9;10;11;16;18
Hu et al. (28)	Cross-sectional study	2024	Shandong, China	Single-center	≥18	305	37.70	FoP-Q-SF	35.30 ± 4.80	36.07	1;2;9;10;18;21;25
Huang et al. (29)	Cross-sectional study	2024	Shandong, China	Single-center	≥18	150	42.66	FoP-Q-SF	21.03 ± 6.30	NA	22
Tang Q et al. (30)	Cross-sectional study	2024	Guangxi, China	Multicenter	≥18	240	38.33	FoP-Q-SF	32.70 ± 3.50	38.70*	1;8;13;18;24
Zhu et al. (31)	Cross-sectional study	2024	Sichuan, China	Multicenter	≥60	320	NA	FoP-Q-SF	34.31 ± 8.39	28.43*	3;6;8;16;25
Cao M (32)	Cross-sectional study	2024	Shandong, China	Multicenter	≥18	214	44.39	FoP-Q-SF	32.75 ± 8.01	37.85	1;9;10;11
Qu (37)	Cross-sectional study	2024	Liaoning, China	Single-center	≥18	241	33.19	FoP-Q-SF	28.77 ± 8.45	31.54	16;26;27
Chen et al. (33)	Cross-sectional study	2025	Henan, China	Multicenter	≥18	277	40.43	FoP-Q-SF	23.91 ± 7.66	NA	23
Tian et al. (34)	Cross-sectional study	2025	Hebei, China	Multicenter	≥60	248	46.77	FoP-Q-SF	28.43 ± 0.47	23.40	17;18;19
Li et al. (35)	Cross-sectional study	2025	Jiangsu, China	Single-center	≥18	246	52.43	FoP-Q-SF	29.52 ± 7.03	NA	22
Ma et al. (36)	Cross-sectional study	2025	Shaanxi, China	Single-center	≥60	203	51.72	FoP-Q-SF	32.98 ± 10.15	47.78	7;16;18;23
Sun et al. (38)	Cohort study	2025	Jiangsu, China	Single-center	≥18	154	NA	FoP-Q-SF	37.19 ± 6.72	40.90*	1;7;9;14;15;16;18
Tang Y. et al. (39)	Cross-sectional study	2025	Anhui, China	Multicenter	≥18	362	43.37	FoP-Q-SF	32.70 ± 10.20	NA	23
Xu et al. (40)	Cross-sectional study	2025	Shandong, China	Single-center	≥18	120	41.66	FoP-Q-SF	30.58 ± 3.51	34.17	4;7;8;10;28;29

1. *Patients were classified into the high-FoP group using LPA or LGM.

2. NA, Not available.

3. Associated Factors: 1. Age, 2. Gender, 3. Residence, 4. Education level, 5. Household income level, 6. Health insurance type, 7. Number of hospitalizations, 8. Comorbidity count, 9. NYHA functional classification, 10. Disease duration, 11. LVEF, 12. Number of oral medications, 13. New-onset HF, 14. Depression, 15. Anxiety, 16. Self-efficacy, 17. Illness perception, 18. Social support, 19. Psychological resilience, 20. Self-care confidence, 21. Level of self-disclosure, 22. Sleep quality, 23. Quality of life, 24. Health literacy, 25. Symptom perception, 26. Perceived family support, 27. Meaning in life, 28. Activities of daily living, 29. Coping style.

### 3.3 FoP assessment tools

All included studies used the Fear of Progression Questionnaire-Short Form (FoP-Q-SF) to assess FoP. The FoP-Q-SF was adapted from the original Fear of Progression Questionnaire (FoP-Q). It has been applied in patients with various chronic conditions, including cancer, diabetes, and heart disease (41, 42), and is widely validated as a unidimensional measure (43, 44). In 2015, Wu Qiyun translated the FoP-Q-SF into Chinese (45). However, the Chinese version comprises 12 items across two dimensions, namely physical health and social/family functioning. It uses a 5-point Likert scale, with responses ranging from 1 (never) to 5 (always). Scores range from 12 to 60. A score of 34 or higher suggests clinically significant FoP, and a higher score indicates more severe FoP (33, 46). The Chinese version showed good reliability, with Cronbach’s α values of 0.883 for the total scale, 0.829 for the physical health dimension, and 0.812 for the social/family dimension.

### 3.4 Current status of FoP in HF

All included studies reported the mean FoP-Q-SF scores in patients with HF, which ranged from 21.03 to 37.19. Among these, eight studies reported scores for the two dimensions of the FoP-Q-SF. Seven studies (18, 25, 28, 32, 35, 37, 39) showed that patients scored higher on the physical health dimension than on the social/family dimension, whereas one study (29) reported the opposite finding. Ten studies (18, 25–28, 32, 34, 36, 37, 40) indicated that the proportion of patients with FoP-Q-SF scores of ≥34 ranged from 23.40% to 48.00%. Three additional studies used latent variable approaches to examine FoP, including latent profile analysis (LPA) in two studies (30, 31) and latent growth model (LGM) in one study (38). The proportion of patients classified into the high-FoP group ranged from 28.43% to 40.90%. Two studies (18, 38) conducted follow-up assessments at 3 and 6 months post-discharge and found that FoP levels gradually decreased over time. Three studies (18, 25, 32) showed that higher FoP-Q-SF scores were consistently associated with the item “Worry about what would happen to my family if something happened to me”.

### 3.5 Associated factors of FoP in HF

**Figure 3:**
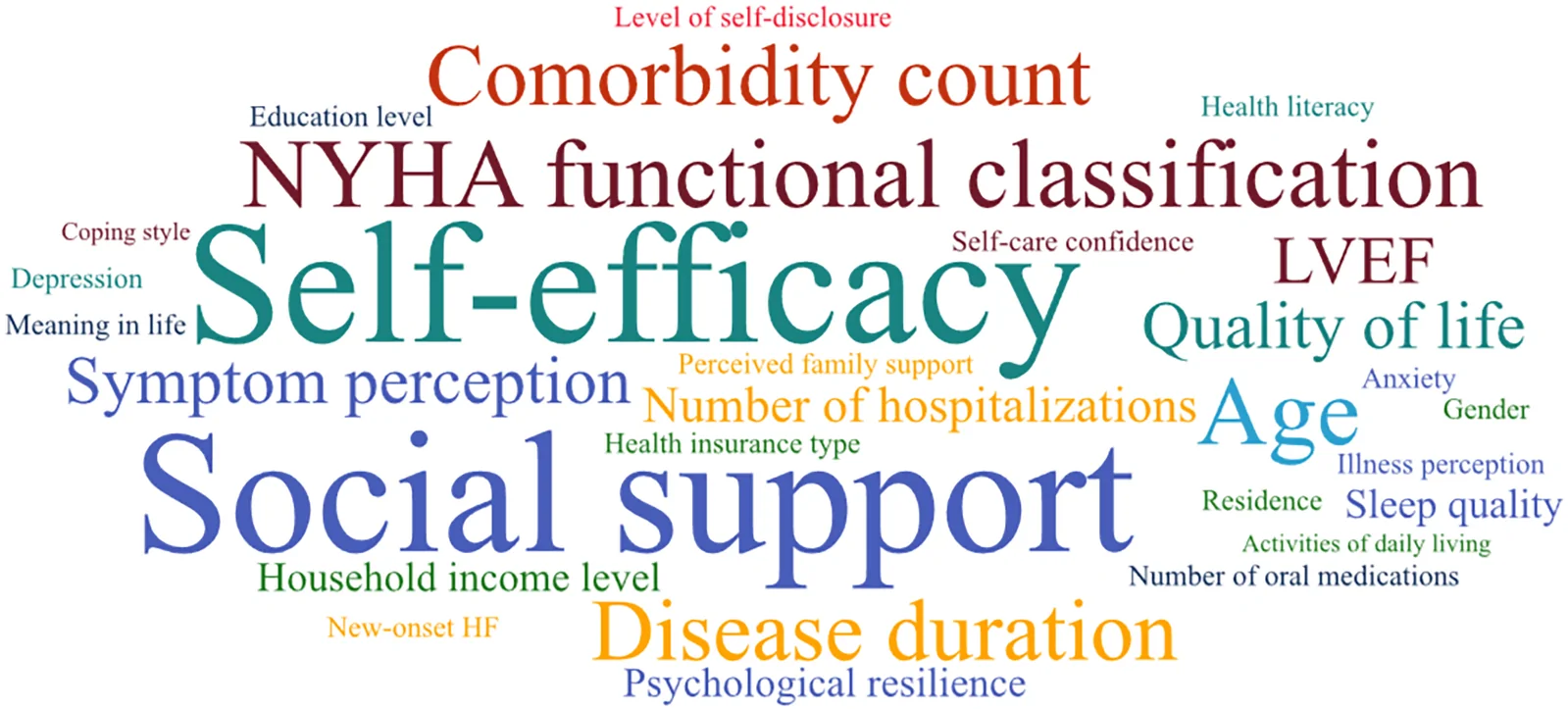
Word cloud of factors associated with FoP in patients with HF. Word size reflects the frequency of occurrence of each factor.

**Figure 4:**
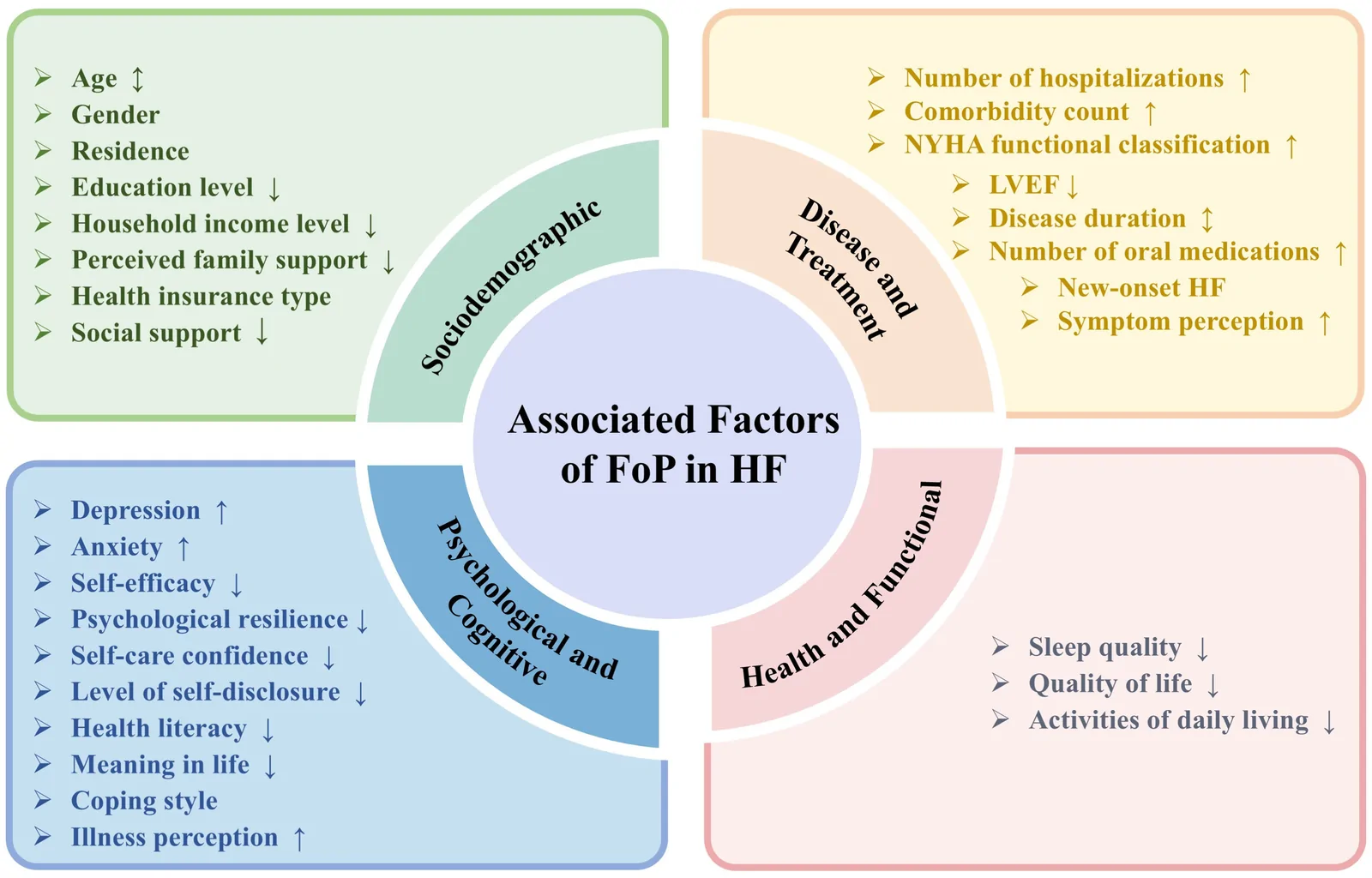
Factors associated with FoP in patients with HF. Arrows indicate the direction of association with FoP (↑ Positive association; ↓ Negative association; ↕ Mixed findings (both positive and negative associations reported).

Twenty-nine factors associated with FoP in patients with HF were identified from the included studies and grouped into four main categories, as shown in **Figures 3**, **4** and **Table 2**.

**Table 2:** Comprehensive mapping table of associated factors and included studies regarding FoP in HF.

Factor category	Associated factors	Included studies	
		Positive association	Negative association
Sociodemographic factors	Age	Sun et al. (38)	Xiong (18); Hu et al. (28); Tang Q et al. (30); Cao M (32)
	Gender	Hu et al. (28)	
	Residence	Zhu et al. (31)	
	Education level		Xu et al. (40)
	Household income level		Zhao et al. (27)
	Perceived family support		Qu (37)
	Health insurance type	Zhu et al. (31)	
	Social support		Du et al. (25); Zhao et al. (27); Hu et al. (28); Tang Q et al. (30); Tian et al. (34); Ma et al. (36); Sun et al. (38)
Disease and treatment factors	Number of hospitalizations	Ma et al. (36); Sun et al. (38); Xu et al. (40)	
	Comorbidity count	Zhao et al. (27); Tang Q et al. (30); Zhu et al. (31); Xu et al. (40)	
	NYHA functional classification	Cao Y et al. (26); Zhao et al. (27); Hu et al. (28); Cao M (32); Sun et al. (38)	
	LVEF		Cao Y et al. (26); Zhao et al. (27); Cao M (32)
	Disease duration	Zhao et al. (27); Hu et al. (28); Xu et al. (40)	Cao M (32)
	Number of oral medications	Xiong (18)	
	New-onset HF	Tang Q et al. (30)	
	Symptom perception	Xiong (18); Hu et al. (28); Zhu et al. (31)	
Psychological and cognitive factors	Depression	Sun et al. (38)	
	Anxiety	Sun et al. (38)	
	Self-efficacy		Du et al. (25); Cao Y et al. (26); Zhao et al. (27); Zhu et al. (31); Ma et al. (36); Qu (37); Sun et al. (38)
	Psychological resilience		Xiong (18); Tian et al. (34)
	Self-care confidence		Xiong (18)
	Level of self-disclosure		Hu et al. (28)
	Health literacy		Tang Q et al. (30)
	Meaning in life		Qu (37)
	Coping style	Xu et al. (40)	
	Illness perception	Tian et al. (34)	
Health and functional factors	Sleep quality		Huang et al. (29); Li et al. (35)
	Quality of life		Chen et al. (33); Ma et al. (36); Tang Y et al. (39)
	Activities of daily living		Xu et al. (40)

#### 3.5.1 Sociodemographic factors

Several sociodemographic factors were associated with FoP in patients with HF. Four studies (18, 28, 30, 32) reported higher FoP in middle-aged and younger patients than in older patients, whereas Sun et al. (38) reported the opposite finding. One study (28) found higher FoP in women than in men. Zhu et al. (31) reported that patients living in urban areas had lower FoP. In addition, patients covered by Basic Medical Insurance for Urban Workers had lower FoP than those covered by Basic Medical Insurance for Urban and Rural Residents. Xu et al. (40) found that an education level of junior high school or below was a risk factor for higher FoP. One study (27) indicated that patients with low income had higher FoP. Seven studies (25, 27, 28, 30, 34, 36, 38) showed a significant negative correlation between FoP and social support. Another study (37) similarly found that perceived family support was negatively correlated with FoP.

#### 3.5.2 Disease and treatment factors

Three studies (36, 38, 40) showed that more frequent hospitalizations were associated with higher FoP in patients with HF. Four studies (27, 30, 31, 40) reported a positive correlation between comorbidity burden and FoP. Cardiac function status was associated with FoP severity. Five studies (26–28, 32, 38) found a positive correlation between New York Heart Association (NYHA) functional classification and FoP, and three studies (26, 27, 32) reported higher FoP in patients with reduced left ventricular ejection fraction (LVEF). Disease duration was also associated with FoP. Three studies (27, 28, 40) indicated that FoP increased with longer disease duration, whereas Cao M (32) reported higher FoP in patients with a disease duration of less than one year. Xiong (18) found that a greater number of oral medications was associated with higher FoP, and Tang Q et al. (30) identified new-onset HF as an additional risk factor. Three studies (18, 28, 31) showed that more severe symptom perception, defined as patients’ evaluation of physical symptoms and the associated distress, was associated with higher self-reported FoP.

#### 3.5.3 Psychological and cognitive factors

Among the 17 included studies, Sun et al. (38) observed that elevated levels of depression and anxiety were correlated with higher FoP in patients with HF. Seven studies (25–27, 31, 36–38) demonstrated a significant inverse correlation between FoP and self-efficacy. Psychological resilience was defined as the ability to return to a normal functional state following illness or trauma; two studies (18, 34) showed a negative association between psychological resilience and FoP. Xiong (18) found lower FoP in patients with higher self-care confidence. Hu et al. (28) reported that patients’ self-disclosure level was negatively related to FoP. Tang Q et al. (30) reported that high health literacy was associated with lower FoP. Qu (37) found that a stronger sense of meaning in life was associated with lower FoP. Xu et al. (40) found that a negative coping style was associated with higher FoP. Illness perception, which reflects patients’ cognitive and emotional understanding of illness (47), was also positively correlated with FoP (34).

#### 3.5.4 Health and functional factors

Two studies (29, 35) found that poorer sleep quality reported by patients with HF was associated with more severe FoP. Three studies (33, 36, 39) reported that patients with poorer quality of life had higher FoP. Xu et al. (40) noted that patients with poorer activities of daily living (ADL) and greater care dependency had higher levels of FoP.

### 3.6 Quality assessment results

Based on the JBI Critical Appraisal Checklists, 13 of the 17 included studies achieved a perfect score of 100%, 2 scored 90.9%, and 2 scored 75%. Overall, 15 studies (88.2%) were rated as high quality. Detailed quality assessment results are presented in **Table 3** and **Table 4**.

**Table 3:** Quality assessment of cross−sectional studies based on the JBI Critical Appraisal Checklist.

Items	Du et al. (25)	Cao Y et al. (26)	Zhao et al. (27)	Hu et al. (28)	Huang et al. (29)	Tang Q et al. (30)	Zhu et al. (31)	Cao M (32)	Chen et al. (33)	Tian et al. (34)	Li et al. (35)	Ma et al. (36)	Qu (37)	Tang Y et al. (39)	Xu et al. (40)
1. Were the criteria for inclusion in the sample clearly defined?	YES	YES	YES	YES	YES	YES	YES	YES	YES	YES	YES	YES	YES	YES	YES
2. Were the study subjects and the setting described in detail?	YES	YES	YES	YES	YES	YES	YES	YES	YES	YES	YES	YES	YES	YES	YES
3. Was the exposure measured in a valid and reliable way?	YES	YES	YES	YES	YES	YES	YES	YES	YES	YES	YES	YES	YES	YES	YES
4. Were objective, standard criteria used for measurement of the condition?	YES	YES	YES	YES	YES	YES	YES	YES	YES	YES	YES	YES	YES	YES	YES
5. Were confounding factors identified?	NO	YES	YES	YES	YES	YES	YES	YES	YES	YES	YES	YES	YES	NO	YES
6. Were strategies to deal with confounding factors stated?	NO	YES	YES	YES	YES	YES	YES	YES	YES	YES	YES	YES	YES	NO	YES
7. Were the outcomes measured in a valid and reliable way?	YES	YES	YES	YES	YES	YES	YES	YES	YES	YES	YES	YES	YES	YES	YES
8. Was appropriate statistical analysis used?	YES	YES	YES	YES	YES	YES	YES	YES	YES	YES	YES	YES	YES	YES	YES
Total score	6	8	8	8	8	8	8	8	8	8	8	8	8	6	8
Percentage of maximum score	75%	100%	100%	100%	100%	100%	100%	100%	100%	100%	100%	100%	100%	75%	100%

**Table 4:** Quality assessment of cohort studies based on the JBI Critical Appraisal Checklist.

Items	Xiong (18)	Sun et al. (38)
1. Were the two groups similar and recruited from the same population?	YES	YES
2. Were the exposures measured similarly to assign people to both exposed and unexposed groups?	YES	YES
3. Was the exposure measured in a valid and reliable way?	YES	YES
4. Were confounding factors identified?	YES	YES
5. Were strategies to deal with confounding factors stated?	YES	YES
6. Were the groups/participants free of the outcome at the start of the study (or at the moment of exposure)?	YES	YES
7. Were the outcomes measured in a valid and reliable way?	YES	YES
8. Was the follow up time reported and sufficient to be long enough for outcomes to occur?	YES	YES
9. Was follow up complete, and if not, were the reasons to loss to follow up described and explored?	YES	YES
10. Were strategies to address incomplete follow up utilized?	NO	NOT APPLICABLE
11. Was appropriate statistical analysis used?	YES	YES
Total score	10	10
Percentage of maximum score	90.9%	90.9%

## 4 Discussion

The negative clinical consequences of FoP in patients with HF are governed by several underlying mechanisms. Firstly, FoP acts as a persistent psychological stressor capable of hyperactivating the sympathetic nervous system and the hypothalamic-pituitary-adrenal (HPA) axis (48). This physiological cascade ultimately exacerbates systemic inflammation, raises the risk of cardiovascular events, and accelerates HF progression (49). Secondly, persistent high FoP contributes to maladaptive avoidance behaviors, most notably kinesiophobia. Holding the false belief that physical activity or work-related strain can trigger HF exacerbation or sudden cardiac death, patients voluntarily limit physical participation. This behavioral restriction constrains routine daily living, prolongs work absence, and accelerates physical deconditioning (22). Thirdly, high FoP leads to somatic amplification and catastrophic thinking (50). Patients tend to mistake normal bodily sensations for HF progression. This psychological burden leads to poor treatment adherence, as patients may intentionally skip medications to avoid illness reminders or misinterpret side effects as a worsening condition (15). Such constant hypervigilance impairs quality of life and increases mental distress. Furthermore, excessive worry about physical symptoms drives unnecessary healthcare utilization, resulting in frequent unplanned medical visits.

Given these complex mechanisms and their adverse impacts, a comprehensive understanding of current FoP research is essential. Therefore, this scoping review synthesized evidence from 17 eligible studies and provided a comprehensive overview of FoP assessment tools, prevalence, and associated factors among patients with HF. The main findings are as follows:

### 4.1 Limited availability of HF-specific FoP assessment tools

All studies included in this scoping review employed the FoP-Q-SF to measure FoP. Although this may have enhanced the consistency of findings to some extent, it also highlights the current shortage of disease-specific assessment tools for this population. The development of FoP-related assessment tools for cardiac populations remains limited. In 2021, Li developed the FoP scale for patients with acute myocardial infarction, comprising 17 items across three dimensions (51). In 2025, Clarke et al. developed the Cardiac Recurrence and Progression Fear Scale and applied it in patients with heart disease (52, 53). As of December 2025, no dedicated assessment tools have been developed specifically for FoP in patients with HF.

### 4.2 Common occurrence of FoP and prominent concerns about physical health

FoP-Q-SF scores in patients with HF ranged from 21.03 to 37.19, with an upper range lower than that reported in patients with breast cancer (23.57–45.79) (54). Meanwhile, the prevalence of FoP in patients with HF ranged from 23.40% to 48.00%, with nearly one-third of the studies reporting a prevalence exceeding 40%, which is substantially higher than the 23.10% to 35.80% range reported in diabetic populations (55, 56). These findings suggest that FoP is a common psychological concern among patients with HF in the available Chinese literature, warranting greater attention from clinicians and researchers. Patients with HF scored higher on the physical health dimension than on the social/family dimension of the FoP-Q-SF, indicating greater concern about disease progression and treatment-related risks. A similar pattern has been reported in gastric cancer research (57). This dimensional difference may be explained by the progressive nature of HF. Symptoms such as chest tightness, dyspnea, and fatigue may gradually become worse, affecting daily functioning and overall health, and lead to psychological distress (58). The level of FoP gradually declines with time since discharge, which may be attributed to better adaptation to the disease and its treatment, alleviation of physical symptoms, stabilization of psychological status, improved self-management abilities, and greater perceived control over the disease (59). Patients scored higher on the FoP-Q-SF item “Worry about what would happen to my family if something happened to me,” likely because of their central role in the family and concerns about not fulfilling family responsibilities or placing a greater burden on relatives after disease onset. Accordingly, healthcare professionals should routinely monitor FoP upon admission and at 3 and 6 months post-discharge to identify psychological distress early and enable tailored interventions. Such screening is particularly crucial for patients with high symptom burden or central family roles. Meanwhile, optimizing physical symptom management and self-management education during these follow-ups can enhance patients’ perceived control over the disease and alleviate FoP.

### 4.3 Multidimensional factors associated with FoP in patients with HF

#### 4.3.1 Sociodemographic factors

Most studies indicated that younger and middle-aged patients with HF tend to report higher levels of FoP, similar to breast cancer patients (54), likely due to their significant family and work responsibilities, combined with relatively limited life experience and lower psychological resilience (60). Adequate social support can help patients reintegrate into the workforce while maintaining and improving their overall physical and mental health. Emotional support from family, friends, and social networks, in particular, provides comfort and understanding and may help alleviate feelings of loneliness and anxiety (61). Accordingly, social support may help alleviate FoP in this patient population, which is consistent with findings in patients with multiple sclerosis (62). Household income is a key determinant of patients’ medical expenses and financial security in daily life. Low-income patients face substantial financial burdens due to prolonged treatment costs and reduced work capacity, which may increase their FoP and uncertainty about future health outcomes (63). Therefore, healthcare professionals should prioritize younger and middle-aged patients, those with insufficient social support, and those facing financial hardship by facilitating their access to healthcare resources and support services. For example, individualized health education programs on disease self-management, diet, and exercise can be tailored to patients’ age and income and provided in various formats, such as videos, illustrations, and written materials. Home visits may be arranged when necessary.

#### 4.3.2 Disease and treatment factors

Patients with multiple chronic comorbidities not only face challenges in treatment planning and disease prognosis but also experience more severe physical symptoms, faster disease progression, and a higher risk of adverse treatment reactions and complications (64). These factors may complicate clinical assessment, heighten illness-related concerns (27, 30, 40), and increase the risk of FoP. Similar associations between a higher comorbidity burden and elevated FoP have also been reported in patients with diabetes (55). NYHA functional class and LVEF are key indicators of HF severity. A higher NYHA functional class or lower LVEF indicates more severe disease and more pronounced symptoms such as dyspnea and fatigue. Daily life and work may also be more limited. More severe disease is often associated with a poorer prognosis, which may increase patients’ uncertainty regarding their condition. This may lead to negative emotions such as anxiety and depression, thereby increasing the likelihood of FoP (26–28, 32, 38). Therefore, healthcare professionals should use online health management platforms for remote monitoring and timely follow-up, which may help detect early signs of worsening, facilitate prompt interventions, reduce acute exacerbations, and improve long-term quality of life and clinical outcomes.

#### 4.3.3 Psychological and cognitive factors

Self-efficacy has received increasing attention in recent research. It refers to an individual’s confidence in their ability to organize and carry out the actions needed to achieve their goals (65). Patients with higher self-efficacy can better cope with disease-related stress, adopt active disease management strategies, and maintain a stronger sense of control over their illness. This may help them better adjust to illness perceptions and emotional states and reduce FoP to some extent (66). By contrast, patients with low self-efficacy may interpret disease-related information more negatively and make less use of available social support, which may lead to higher FoP. Therefore, healthcare professionals should assess and enhance patients’ self-efficacy at an early stage to help alleviate FoP.

#### 4.3.4 Health and functional factors

Patients with poor quality of life often have weakened cardiac function and severe physical symptoms. These factors keep them under long-term psychological stress and may increase their FoP. Therefore, healthcare professionals should provide targeted health education and encourage these patients to adopt proactive coping strategies, which can help reduce psychological distress and improve overall mental well-being.

#### 4.3.5 Interrelationships among factors associated with FoP

Existing literature suggests that the identified factors are closely connected rather than acting independently. For example, patients’ sociodemographic characteristics, clinical conditions, and quality of life are all linked to their psychological states. Effective social support not only improves psychological resilience but may also help reduce anxiety and depression to some extent (67). Furthermore, educational level and disease self-management may significantly affect patients’ self-efficacy (68). Therefore, HF management should include comprehensive evaluations of patients’ social, psychological, clinical, and functional status, together with individualized interventions to reduce FoP and support overall physical and mental health.

### 4.4 Clinical implications

While FoP is a prevalent psychological burden across various chronic diseases, its manifestations differ markedly by disease trajectory. Patients with cancer or diabetes tend to have stable worries about foreseeable adverse outcomes. Cancer patients fear tumor recurrence and metastasis, while diabetic patients focus on the strict lifelong medical management (56). These worries are strongly associated with slow disease progression or treatment-related adverse effects. In contrast, FoP in HF arises from unpredictable cardiac dysfunction and the risk of abrupt acute decompensation (69). This unpredictability triggers prominent somatic hypervigilance, causing patients to often misread mild dyspnea or fatigue as signals of rapid cardiac deterioration or impending death. This distinction suggests that FoP in HF is a dynamic, symptom-driven psychological state rather than a static fear of a discrete event. In this context, the findings of this scoping review offer significant clinical implications. First, this study systematically identified and synthesized 29 factors associated with FoP in patients with HF. These findings can help identify patients at high risk early and support targeted psychological interventions in clinical practice. Second, the results highlighted that FoP arises from the interaction of social, clinical, and psychological factors, providing a theoretical foundation for future research to design multicomponent interventions targeting FoP. Finally, this scoping review may support a shift in HF care from a purely physiological focus toward an approach that better integrates psychological and social rehabilitation.

### 4.5 Limitations of the study

First, most studies included in this scoping review were cross-sectional (88.23%), which limited the ability to capture changes in FoP over time. Second, this scoping review included only formally published studies in Chinese and English and did not search for gray literature, which may have resulted in the omission of real-world clinical intervention experiences and introduced potential publication bias. Third, all included studies were conducted in China, which may reflect cross-country differences in attention to FoP among patients with HF. Therefore, caution is warranted when generalizing these findings to populations in other countries or cultural contexts. Fourth, compared with a meta-analysis, this scoping review did not provide pooled effect sizes, which may limit the direct applicability of the findings to clinical practice.

### 4.6 Directions for future studies

Future research should utilize longitudinal designs to focus on the dynamic patterns of FoP in patients with HF across different HF stages and social contexts, and to explore its causal relationships with morbidity, prognosis, and health-related quality of life (HRQoL). Secondly, well-designed intervention studies should be undertaken to develop and evaluate targeted nursing strategies that aim to reduce FoP in this population and provide evidence-based support for integrated psychological care. Finally, while the FoP-Q-SF is useful for cross-study comparisons, it lacks sensitivity to HF-specific concerns, such as acute decompensation, sudden cardiac death, readmissions, and declining cardiac function. Therefore, developing HF-specific tools is essential to enhance screening and risk stratification, yet this approach demands extensive validation and inevitably limits cross-disease comparability.

## 5 Conclusion

This scoping review shows that FoP is relatively common among patients with HF and is influenced by factors in four main areas: sociodemographic characteristics, disease and treatment conditions, psychological and cognitive factors, and health and functional status. These findings may help healthcare professionals better identify patients at high risk of FoP and promote HF care that goes beyond physical rehabilitation to include psychological and social rehabilitation. Future research should focus on understanding how FoP changes over time and identifying effective interventions. In addition, assessment tools specific to HF should be developed to support both clinical practice and research in this field. Finally, studies across broader geographical and cultural contexts are needed to validate and expand upon these findings.
