# Supplementary material for: Prevalence and associated factors of fear of progression in patients with heart failure in China: a scoping review
Source: Front Psychiatry. 2026 Aug 6;17:1865683. doi: 10.3389/fpsyt.2026.1865683 (PMC13492199; doi:10.3389/fpsyt.2026.1865683)
Supplement: Supplementary file 1 [file Table1.docx]

Supplementary Material

# 1 Search Queries for Each Database:

The search covered studies published from the start of each database to December 31, 2025, and the final search was conducted on January 31, 2026.

## 1.1 PubMed: 548 records were identified.

(((fear[MeSH Terms]) OR ((Fear*[Title/Abstract]) OR (Cue, Threat[Title/Abstract]) OR (Threat Cue*[Title/Abstract]) OR (Threat Sensitivit*[Title/Abstract]) OR (Sensitivity, Threat[Title/Abstract]))) OR ((Fear of Recurrence[Title/Abstract]) OR (Fear of progression[Title/Abstract]) OR (Disease progression fear[Title/Abstract]) OR (FOP[Title/Abstract]) OR (Fear of Relapse[Title/Abstract]))) AND ((Heart Failure[MeSH Terms]) OR (Heart Failure[Title/Abstract]) OR (Cardiac Failure[Title/Abstract]) OR (Heart Decompensation[Title/Abstract]) OR (Congestive Heart Failure[Title/Abstract]) OR (Myocardial Failure[Title/Abstract]))

## 1.2 Web of Science Core Collection: 718 records were identified.

#1: AB=("Fear*" OR "Cue, Threat" OR "Threat Cue*" OR "Threat Sensitivit*" OR "Sensitivity, Threat" OR "Fear of progression" OR "Disease progression fear" OR "FOP" OR "Fear of Recurrence" OR "Fear of Relapse")

#2: TS=("Heart Failure" OR "Cardiac Failure" OR "Heart Decompensation" OR "Congestive Heart Failure" OR "Myocardial Failure")

#3: #1 AND #2

## 1.3 Scopus: 245 records were identified.

TITLE-ABS-KEY("Fear*" OR "Cue, Threat" OR "Threat Cue*" OR "Threat Sensitivit*" OR "Sensitivity, Threat" OR "Fear of progression" OR "Disease progression fear" OR "FOP" OR "Fear of Recurrence" OR "Fear of Relapse") AND TITLE("Heart Failure" OR "Cardiac Failure" OR "Heart Decompensation" OR "Congestive Heart Failure" OR "Myocardial Failure")

## 1.4 Embase: 374 records were identified.

('heart failure':ti OR 'cardiac failure':ti OR 'heart decompensation':ti OR 'congestive heart failure':ti OR 'myocardial failure':ti) AND ('fear*':ti,ab,kw OR 'cue, threat':ti,ab,kw OR 'threat cue*':ti,ab,kw OR 'threat sensitivit*':ti,ab,kw OR 'sensitivity, threat':ti,ab,kw OR 'fear of progression':ti,ab,kw OR 'disease progression fear':ti,ab,kw OR 'fop':ti,ab,kw OR 'fear of recurrence':ti,ab,kw OR 'fear of relapse':ti,ab,kw) AND [1966-2025]/py

## 1.5 CINAHL: 131 records were identified.

SU ("Heart Failure" OR "Cardiac Failure" OR "Heart Decompensation" OR "Congestive Heart Failure" OR "Myocardial Failure") AND XB ("Fear*" OR "Cue, Threat" OR "Threat Cue*" OR "Threat Sensitivit*" OR "Sensitivity, Threat" OR "Fear of progression" OR "Disease progression fear" OR "FOP" OR "Fear of Recurrence" OR "Fear of Relapse")

## 1.6 CNKI： 230 records were identified.

(TKA=恐惧 + 害怕 + 恐惧复发 + 恐惧疾病进展 + 疾病进展恐惧 + 疾病恐惧进展) AND (SU=心力衰竭 + 慢性心衰竭 + 慢性心脏功能衰竭 + 慢性心力衰+ 心功能不全 + 心衰 + cardiac failure + heart failure)

## 1.7 Wanfang: 422 records were identified.

(关键词=心力衰竭 OR 慢性心衰竭 OR 慢性心脏功能衰竭 OR 慢性心力衰+ 心功能不全 OR 心衰 OR cardiac failure OR heart failure) AND (摘要=恐惧 OR 害怕 OR 恐惧复发 OR 恐惧疾病进展 OR 疾病进展恐惧 OR 疾病恐惧进展)

## 1.8 VIP: 157 records were identified.

(M=心力衰竭 + 慢性心衰竭 + 慢性心脏功能衰竭 + 慢性心力衰+ 心功能不全 + 心衰 + cardiac failure + heart failure) AND (U=恐惧 OR 害怕 OR 恐惧复发 OR 恐惧疾病进展 OR 疾病进展恐惧 OR 疾病恐惧进展)

## 1.9 CBM: 29 records were identified.

(标题=心力衰竭 OR 慢性心衰竭 OR 慢性心脏功能衰竭 OR 慢性心力衰+ 心功能不全 OR 心衰 OR cardiac failure OR heart failure) AND (摘要=恐惧 OR 害怕 OR 恐惧复发 OR 恐惧疾病进展 OR 疾病进展恐惧 OR 疾病恐惧进展)

## 1.10 CMA Journals Full-text Database: 21 records were identified.

(关键词=心力衰竭 OR 慢性心衰竭 OR 慢性心脏功能衰竭 OR 慢性心力衰+ 心功能不全 OR 心衰 OR cardiac failure OR heart failure) AND (所有=恐惧 OR 害怕 OR 恐惧复发 OR 恐惧疾病进展 OR 疾病进展恐惧 OR 疾病恐惧进展)
